# Micro- and Nanocellulose in Polymer Composite Materials: A Review

**DOI:** 10.3390/polym13020231

**Published:** 2021-01-11

**Authors:** Abdoulhdi A. Borhana Omran, Abdulrahman A. B. A. Mohammed, S. M. Sapuan, R. A. Ilyas, M. R. M. Asyraf, Seyed Saeid Rahimian Koloor, Michal Petrů

**Affiliations:** 1 Department of Mechanical Engineering, College of Engineering, Universiti Tenaga Nasional, Jalan Ikram-Uniten, Kajang 43000, Selangor, Malaysia; rahman.aziz@uniten.edu.my; 2 Department of Mechanical Engineering, College of Engineering Science & Technology, Sebha University, Sabha 00218, Libya; 3 Laboratory of Biocomposite Technology, Institute of Tropical Forestry and Forest Products (INTROP), Universiti Putra Malaysia, Serdang 43400, Selangor, Malaysia; 4 Advanced Engineering Materials and Composites Research Centre (AEMC), Department of Mechanical and Manufacturing Engineering, Universiti Putra Malaysia, Serdang 43400, Selangor, Malaysia; 5 Sustainable Waste Management Research Group (SWAM), School of Chemical and Energy Engineering, Faculty of Engineering, Universiti Teknologi Malaysia, Johor Bahru 81310, Johor, Malaysia; 6 Centre for Advanced Composite Materials, Universiti Teknologi Malaysia, Johor Bahru 81310, Johor, Malaysia; 7 Department of Aerospace Engineering, Universiti Putra Malaysia, Serdang 43400, Selangor, Malaysia; asyrafriz96@gmail.com; 8 Institute for Nanomaterials, Advanced Technologies and Innovation, Technical University of Liberec, Studentská 2, 461 17 Liberec, Czech Republic; seyed.rahimian@tul.cz (S.S.R.K.); michal.petru@tul.cz (M.P.)

**Keywords:** natural fiber, nanocellulose, microcellulose, biocomposite, nanocomposite, biopolymer, synthetic polymer

## Abstract

The high demand for plastic and polymeric materials which keeps rising every year makes them important industries, for which sustainability is a crucial aspect to be taken into account. Therefore, it becomes a requirement to makes it a clean and eco-friendly industry. Cellulose creates an excellent opportunity to minimize the effect of non-degradable materials by using it as a filler for either a synthesis matrix or a natural starch matrix. It is the primary substance in the walls of plant cells, helping plants to remain stiff and upright, and can be found in plant sources, agriculture waste, animals, and bacterial pellicle. In this review, we discussed the recent research development and studies in the field of biocomposites that focused on the techniques of extracting micro- and nanocellulose, treatment and modification of cellulose, classification, and applications of cellulose. In addition, this review paper looked inward on how the reinforcement of micro- and nanocellulose can yield a material with improved performance. This article featured the performances, limitations, and possible areas of improvement to fit into the broader range of engineering applications.

## 1. Introduction

Petroleum-based synthesis polymers are non-degradable, with production, recycling, and disposal releasing toxic emissions into the environment [1]. Cellulose offers excellent properties to minimize this damage by utilization as a filler in the manufacturing of either a synthesis matrix or a natural starch matrix. Cellulose is the main substance of a plant’s cell walls, helping plants to remain stiff and upright, hence, it can be extracted from plant sources, agriculture waste, animals, and bacterial pellicle [2,3]. It is composed of polymer chains consisting of unbranched β (1,4) linked D glucopyranosyl units (anhydroglucose unit, AGU) [4,5]. Cellulose also possesses excellent mechanical properties, such as tensile and flexural strengths, tensile and flexural moduli, and thermal resistance, as well as low cost, due to its availability from different resources and abundance in nature, and degradability which is not obtainable in synthetic fillers, that makes it an excellent bio-filler for both synthesis or natural polymer matrixes [6]. Cellulose needs to be extracted to be a useful substance. Cellulose extraction can be achieved via three approaches; mechanical, chemical, and bacterial techniques. Mechanical cellulose extraction comprises of high-pressurized homogenization [7], grinding [8], crushing [9], and steam explosion methods [10]. Chemical extraction methods include alkali treatment [11], acid retting, chemical retting [12], and degumming [13].

**Figure 1:**
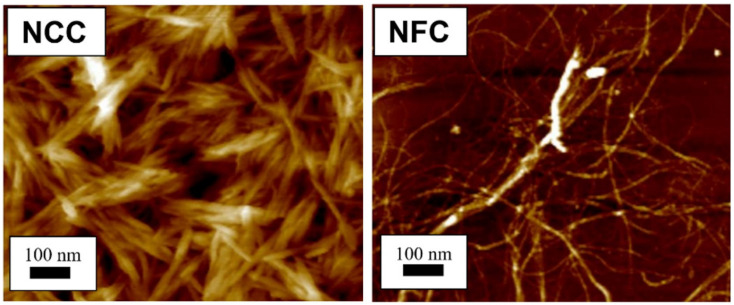
Atomic force microscopy images show different structure between nanocrystalline cellulose (NCC) [23] and nanofibrillated cellulose (NFC) [24]. (Reproduced with copyright permission from Ilyas et al. [23,24]).

Cellulose can be extracted in different sizes, depending on the intended application. Micro- and nanocellulose are the common sizes of cellulose used in industrial applications. Nanocellulose is divided into three types, (1) nanofibrillated cellulose (NFC), also known as nanofibrils or microfibrils or macrofibrillated cellulose or nanofibrillated cellulose; (2) nanocrystalline cellulose (NCC), also known as crystallites, whiskers, or rod-like cellulose microcrystals, and (3) bacterial nanocellulose (BNC), also known as microbial cellulose or biocellulose [14,15]. The difference between microfibrillated cellulose and nanocrystalline cellulose is the fiber size distributions that are wide in microfibrillated cellulose and narrow or drastically shorter in nanocrystalline cellulose [16]. Figure 1 depicts the structural difference between nanofibrillated cellulose and nanocrystalline cellulose. Similar to microfibrillated cellulose, bacterial cellulose also has a narrow size distribution and high crystallinity, except for its source, which is bacteria. According to Alain Dufresne [17] and Chirayil et al. [18], NCC and NFC are renowned not only for their biodegradation, superb properties, unique structures, low density, excellent mechanical performance, high surface area and aspect ratio, biocompatibility, and natural abundance, but also for their possibility to modify their surfaces to enhance their nano-reinforcement compatibility with other polymers due to the presence of abundant hydroxyl groups. Nanocellulose-based materials, also known as a new ageless bionanomaterial, are non-toxic, recyclable, sustainable, and carbon-neutral [17]. NCC and NFC have demonstrated numerous advanced applications, including in the automotive industry, optically transparent materials, drug supply, coating films, tissue technology, biomimetic materials, aerogels, sensors, three-dimensional (3D) printing, rheology modifiers, energy harvesters, filtration, textiles, printed and flexible electronics, composites, paper and board, packaging, oil and gas, medical and healthcare, and scaffolding [19,20]. In addition, macro and mesoporous nanocellulose beads also are utilized in energy storage devices. The cellulose beads act as electrodes that serve as complements to conventional supercapacitors and batteries [21], and depend on the properties of the cellulose (e.g., origin, porosity, pore distribution, pore-size distribution, and crystallinity) [22]. In consequence, the number of patents and publications on nanocellulose over 20 years have increased significantly from 764 in 2000 to 18,418 in 2020. In addition, this increment of more than 2300% over 20 years indicates that nanocellulose has become the advanced emerging material in the 21st century.

Applications of cellulose are vast and interfere with many fields concentrated on mechanical, medical, and industrial applications [25]. In industry, cellulose is used as a filler for matrixes in the manufacturing of a degradable polymer. Cellulose is also used in packaging applications, tissue engineering applications, electronic, optical, sensor, pharmaceutical applications, cosmetic applications, insulation, water filtration, hygienic applications, as well as vascular graft applications [26,27,28]. For instance, in Li-ion battery application, cellulose has been applied along with carbon nanotubes (CNT) as current collectors [29]. Previously, the current collector in the battery used the conventional aluminum foil. From this point of view, cellulose paper-CNTs-based electrodes showed ~17% improvement in areal capacity compared to commercial aluminum-based electrodes. Another renowned application of cellulose is the implementation of electrospun cellulose acetate nanofibers for antimicrobial activity as mentioned by Kalwar and Shen [30]. Moreover, cellulose is highly efficient in antitumor drug delivery [31]. In this case, the application of carboxymethyl cellulose-grafted graphene oxide drug delivery system has a huge potential in colon cancer therapy. The cellulose can also be implemented in the oil and gas industry due to its large surface areas and high volume concentrations along with unique mechanical, chemical, thermal, and magnetic properties [32]. Cellulose can also be used as additive and reinforcement for cross arm application in transmission towers in order to improve their mechanical properties and electrical resistance performance [33,34]. To increase the base of potential applications, cellulose’s properties need to be more flexible in terms of modification and improvement to match the required properties of various applications [35]. In this paper, we focused on the techniques of extracting micro- and nanocellulose, treatment and modification of cellulose, classification, and applications of cellulose. Thus, the objective of this paper is to demonstrate the recent state of development in the field of micro and nanocellulose, explain the process of extracting and modifying different types of cellulose, and highlight the properties improvement of cellulose through examples.

## 2. Classification of Cellulose

Cellulose can be classified into two types based on size, microcellulose and nanocellulose, while nanocellulose can be classified in three types: (1) nano- or microfibrillated cellulose (NFC)/(MFC), (2) nanocrystalline cellulose (NCC), and (3) bacterial nanocellulose (BNC) [36,37]. The advantage of extracting or isolating cellulose is that the nanocellulose can be obtained from microcellulose [6,38], producing different cellulose sizes in a compatible procedure.

Nanocellulose can be categorized into the family in nanofibrillated cellulose (NFC), nanocrystalline cellulose (NCC), and bacterial nanocellulose (BNC). The size of nanocellulose ranges from 5 nm to 100 nm [39]. The difference between nanofibrillated cellulose (NFC) and microfibrillated cellulose (MFC) is that NFC is usually produced using a chemical pretreatment followed by a high-pressurized homogenization, while MFC is commonly yielded from chemical treatment [40]. The sources of NFC or MFC are wood, sugar beet, potato tuber, hemp, and flax. The average diameter is 20–50 nm [41,42]. Meanwhile, for nanocrystalline cellulose (NCC), the average range of NCC diameter and length are 5–70 nm and 100 nm, respectively [43]. NCC can be extracted from several sources like plants (wood, cotton, hemp, flax, wheat straw, mulberry bark, ramie, avicel, and tunicin), algae and bacteria, and animals (tunicates) [44]. Another type of nanocellulose that can be produced from non-plant sources is bacterial nanocellulose (BNC). Using microorganisms in the industry of biopolymers is vital because such microorganisms exhibit rapid growth, allowing for high yields and year-round availability of the product [45]. There are two main methods for producing BNC using microorganisms: static culture and stirred culture [46]. Static culture employs the accumulation of a thick, leather-like white BNC pellicle at the air-liquid interface. The stirred culture synthesizes cellulose in a dispersed manner in the culture medium, forming irregular pellets or suspended fibers [47].

It is better to produce bacterial cellulose by static culture because previous studies have shown that bacterial cellulose produced from a static culture has higher mechanical strength and yields than those obtained from stirred culture. Moreover, stirred culture has a higher probability of microorganism mutations, which might affect BNC production. The disadvantage of a static culture is that it takes more time and a larger area of cultivation [48,49,50,51].

## 3. Microcellulose and Nanocellulose Extraction, Treatment, and Modification

Lately, natural fiber biopolymers have been significantly used as alternatives to synthetic polymer which negatively affected the environment [52]. Green composites can be enrolled in many applications, such as automobiles, packaging, construction, building materials, furniture industry, etc. [53,54,55,56,57,58]. Cellulose is the main component of several natural fibers, such as sugarcane bagasse, cotton, cogon grass, flax, hemp, jute, and sisal [59,60,61,62,63,64], and it can also be found in sea animals, bacteria, and fungi. Cellulose can be extracted in microscale with an excessive amount of mineral acids, the crystalline phases at nanometer range [65], with sizes of 10–200 μm [66], and the mean diameter of approximately 44.28 μm [67]. The structure of microcellulose can be divided into microfibrillated cellulose or microcrystalline cellulose; microcrystalline cellulose has higher strength than the microfibrillated cellulose [4]. Cellulose can also be extracted as nanocellulose size of nanocellulose fiber, which generally contains less than 100 nm in diameter and several micrometers in length [68]. Plant natural fiber consists of cellulose and non-cellulose materials such as lignin, hemicellulose, pectin, wax, and other extractives. Therefore, in order to extract cellulose either as micro or nano, the non-cellulose materials must be removed. There are two common methods to remove non-cellulosic materials that were used by researchers, (I) acid chlorite treatment and (II) alkaline treatment [69,70]. Depending on the conditions of extraction process and extraction technique, the crystalline region of the cellulose can significantly vary in size and aspect ratio. This usually results in the types of fibrils, crystalline, and particle sizes (micro- or nano-size). However, they are normally anisometric.

### 3.1. Cellulose Extraction Techniques

There are several types of cellulose production techniques such as mechanical treatment, chemical treatment, combination of chemi-mechanical process, as well as bacterial production of cellulose.

#### 3.1.1. Mechanical Extraction

High-pressurized homogenization is one of the mechanical extraction techniques. High-pressurized homogenization is used for large-scale nanocellulose production by forcing the material through a very narrow channel or orifice using a piston under high pressure of 50–2000 MPa [66]. This is an environmentally friendly method for nanocellulose isolation [71]. However, there is a possibility for the occurrence of mechanical damage to the crystalline structure using this method [72]. Another mechanical technique is grinding. Grinding is used to separate nanocellulose from fiber by applying shear stress on the fiber by rotating grindstones at approximately 1500 rpm [73]. The heat produced by friction during the fibrillation process leads to water evaporation, which improves the extraction process [74]. In addition, crushing is also used to extract cellulose fiber. This method is used to produce microcellulose in frozen places [75]. The size of the produced cellulose ranges between 0.1 and 1 μm. This process can be used as a pretreatment prior to high-pressurized homogenization to yield nanocellulose. Steam explosion is utilized for the extraction of cellulose, which uses a low energy consumption method to extract the cellulose. Although it does not completely remove lignin, it can be considered a pretreatment. After applying this method, the obtained fiber needs mechanical modification.

#### 3.1.2. Chemical Extraction

**Figure 2:**
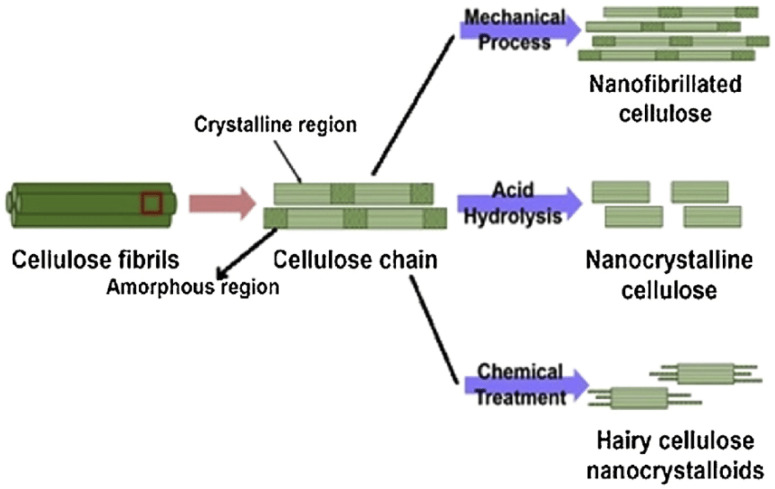
Extraction of nanocellulose from lignocellulosic biomass (reproduced with copyright permission from Sharma et al. [84]).

Chemical extraction procedures extract cellulose by using alkali retting, acid retting, chemical retting, chemical assisted natural (CAN), or degumming to remove the lignin content in the fibers. These treatments also affect other components of the fiber microstructure, including pectin, hemicellulose, and other non-cellulosic materials [76,77,78,79]. One of the examples using the chemical extraction method is alkali or acid retting. This extraction method causes less fiber damage [80], while mechanical extraction is less costly. It is performed by heating, cleaning, and soaking the fiber in alkali or acid solution [81]. This method has the ability to improve some properties of the fiber. Degumming, which is one of the chemical extraction processes that is developed to hold the ramie fiber’s shape, works by eliminating the gummy and pectin content [82]. Another chemical technique is chemical retting. This procedure is used to reduce the lignin and water content in fibers. Chemical retting is able to remove more lignin compared to alkali and acid retting but is less effective in terms of eliminating moisture [12]. A combination of the chemical and mechanical extraction methods can be applied to guarantee higher efficiency of lignin removal, where the mechanical processes usually are done after chemical treatment [83]. Figure 2 shows the extraction of nanocellulose from lignocellulosic biomass via mechanical and chemical methods.

#### 3.1.3. Bacterial Production of Cellulose

Bacterial cellulose is of similar molecular formula to plant origin cellulose, characterized by a crystalline nanofibrillar structure which creates a large surface area that can retain a large amount of liquid. There are many methods for bacterial cellulose preparation, including static, agitated/shaking, and bioreactor cultures. The results of macroscopic morphology, microstructure, mechanical properties of bacterial cellulose are different, depending on the preparation method. The static culture method enhances the accumulation of a gelatinous membrane of cellulose at the surface of the nutrition solution, whereas the agitated/shaking culture affects the asterisk-like, sphere-like, pellet-like, or irregular masses [36]. The required properties and the applications dictate the selection of the appropriate preparation method. Producing cellulose-based bacterial resources gives higher critical surface tension and higher thermal degradation temperature while the cellulose extracted from plants via combination of the chemical and mechanical extraction methods has a hierarchical organization and semi-crystalline nature.

### 3.2. Cellulose Surface Treatment and Modification

Cellulose is the most abundant component that can be found almost exclusively in plant cell walls; it can also be produced by some algae and bacteria [85]. The applications of natural biopolymers have extended in last recent years due to the improvement in the processes of surface treatment and modifications; these applications involve automobiles, construction, building materials like nano building blocks in composites, furniture industry, and optical applications [53,86]. The cellulose fiber has two main drawbacks, (1) high number of hydroxyl groups that makes the product’s structure gel-like and (2) high hydrophilicity [87], which limit its uses in several applications. The purpose of the modification is to improve these two drawbacks to enhance the cellulose’s properties and broaden the applications of natural fiber [88].

To reduce energy consumption during cellulose manufacturing and to extract cellulose in an effective way, pretreatment needs to be carried out. The pretreatment can be either enzymatic pretreatment or TEMPO (2,2,6,6-tetramethylpiperidine-1-oxyl) pretreatment. Enzymatic pretreatment can be divided into cellobiohydrolases and endoglucanases [89,90], which show strong synergistic effects [91]. TEMPO-mediated oxidation pretreatment is a treatment that must be performed in solution. TEMPO-mediated oxidation pretreatment improves the reactivity of cellulose, and the C6 primary hydroxyl groups of cellulose are converted to carboxylate groups via the C6 aldehyde groups [92,93]. The main cellulose modifications are presented in the following subsections.

#### 3.2.1. Molecule Chemical Grafting

The reaction mechanism in this technique is to improve the structure and properties of cellulose, and only happens on the cellulose chains located on the surface of the cellulose. The limitation on the extent of acetylation (ester bonds are formed between cellulose and cyclodextrins) lies in the susceptibility and ease to maintain the surface; however, this technique does not make the cellulose fully dissolved because of the complex network formation [94].

#### 3.2.2. Surface Adsorption on Cellulose

The adsorption on the surface of cellulose is usually done by using surfactants. There are many types of surfactants, such as fluorosurfactant, e.g., perfluorooctadecanoic acid used to coat cellulose [95], cationic surfactant [96,97], and polyelectrolyte solution [9,98]. Surfactants improve hydrophobic behavior; however, they might also change the physical properties and produce some cracks that possibly make absorption of water and moisture occur.

#### 3.2.3. Direct Chemical Modification Methods

The properties of cellulose, such as its hydrophilic or hydrophobic character, elasticity, water sorbency, adsorptive or ion exchange capability, resistance to microbiological attack, and thermal resistance are usually modified by chemical treatments. The main methods of cellulose chemical modification are esterification, etherification, halogenations, oxidation, and alkali treatment [99]. The chemical modifications methods of cellulose are the best methods to achieve adequate structural durability and an efficient adsorption capacity.

#### 3.2.4. Cellulose Grafting

**Figure 3:**
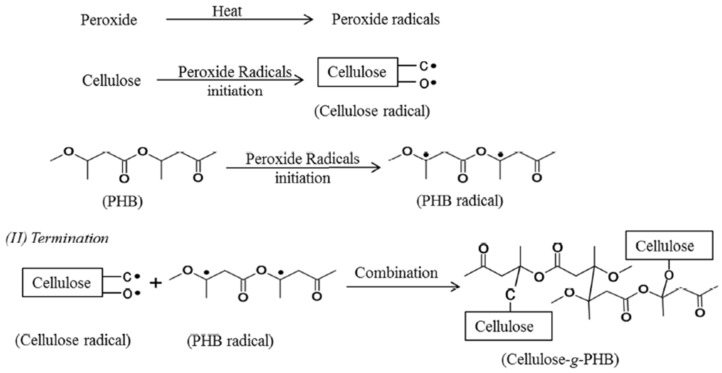
The general mechanism of peroxide radical initiated grafting of polyhydroxybutyrate (PHB) onto cellulose (reproduced with copyright permission from Wei et al. [101]).

Grafting on cellulose by attaching or adding the particles of molecules covalently to the cellulose can be done by either using coupling agents or activating the cellulose substrates [88]. Vinyl monomers grafting on cellulose can be performed in homogeneous or heterogeneous medium [100]. When polyhydroxybutyrate (PHB) is grafted with cellulose, it improves the properties of the cellulose in terms of crystallinity, flexibility, and the chemically linking of the fibers with the matrix [101]. Figure 3 illustrates the general mechanism of peroxide radical initiated grafting of PHB onto cellulose. Polyethylene glycol (PEG) and aminosilane are also used as grafting materials [102,103], where these materials increase the cellulose polarity to have better compatibility with the polymer.

## 4. Mechanical Properties of Microcellulose and Nanocellulose

Developing eco-friendly, sustainable, and easily available materials has gained great attention in recent years, due to environmental issues and the depletion of petroleum. Exploring alternatives for petroleum-based polymers by extracting cellulose from natural resources like plants, animals, and bacteria provides us with an excellent opportunity to save the environment. Nevertheless, this natural cellulosic polymer has some property shortages. Improving its properties extends the abilities of using natural fiber in wider applications. Usually, improving mechanical properties results in enhancements in the other properties, such as thermal and chemical properties. This is due to the modifications applied to the natural fiber improving the homogeneity between the particles, hence creating a good chemical bonding [55]. The important mechanical tests to assess the enhancements resulted from the modifications are tensile strength, tensile modulus, flexural strength, flexural modulus, elongation, and stiffness [104,105].

### 4.1. Tensile Strength

It is essential to know the maximum load that the material can withstand before failure prior to application in any field. Adding microcellulose to polymers has improved the tensile strength of many polymers, e.g., adding microcrystalline cellulose (MCC) prepared from cotton fabric waste to poly(vinyl chloride) film enhanced the tensile strength with the increase of MCC content [106]. When MCC was added to poly(vinyl alcohol), the tensile strength continued to increase with MCC addition. The maximum tensile strength was achieved with 10% of MCC load; beyond that value, the tensile strength started to drop gradually [107]. The composite of polypropylene (PP)/MCC revealed an insignificant decrease in the tensile strength; this problem can be solved by using coupling agents, such as aminopropyltriethoxysilane and maleic anhydride-grafted polypropylene (MAPP). In the case of using coupling agents, the tensile strength was improved, giving a chance to add more MCC [108]. The composite of hydroxypropyl starch/MCC showed improvement in the tensile strength, where the maximum load was 6% of MCC, and the tensile strength started to decrease when adding more MCC [109]. MCC-reinforced polypropylene composites using maleic anhydride polypropylene (MAPP) as a coupling agent improved the tensile strength by 27% higher than that of the composite containing only MAPP [110]. Reinforcing nano-clay with MCC improved the tensile strength of the composite at 7.5 wt% nano-clay [111]. Incorporation of nano-cellulose into gelatin and starch matrices showed that increasing nanocellulose composition to 10% led to increasing the tensile strength [112]. In the composite of poly(vinyl alcohol)/NCC and nanosilica, the addition of NCC and nanosilica to poly(vinyl alcohol) improved the tensile strength [113]. Nanocellulose reinforced unsaturated polyester (UPR) revealed significant increment of tensile strength with the addition of 0.5–3 wt% of NCC with maximum tensile strength obtained for 0.5 wt% [114]. In another study, the composite of chitosan reinforced with NCC improved the tensile strength result until it reached 245 MPa [115].

### 4.2. Flexural Strength

Flexural strength is defined as the maximum bending stress that the material can withstand before yield. Adding modified MCC to cement mortar was found to improve the flexural strength by double [116]. The composite of MCC and cementitious improved the cementitious by 19.2% with 1% of MCC addition [117]. Using nutshell with MCC to fill high-density polyethylene also made remarkable improvements in the flexural strength of the high-density polyethylene [118]. In the composite of polymethylmethacrylate (PMMA) and nanocrystalline cellulose (NCC), the addition of NCC improved the flexural strength to around 3 MPa [119]. The addition of NCC to cement paste showed 20–30% improvement in flexural strength, with only 0.2% of NCC [120,121]. The composite of NCC to epoxy nanocomposites exhibited a 20% enhancement in the flexure strength [122].

### 4.3. Elastic Modulus (Young’s Modulus)

To measure the stiffness of a material, we need to measure the tensile modulus. The addition of MCC to commercial acrylic adhesive increased the modulus of the composition [123]. Solution casting of MCC and organophilic silica (R972) applied as a filler to poly(lactic acid) (PLA) revealed an improvement in the tensile modulus [124]. Polycaprolactone/MCC/wood flour composites exhibited lower tensile modulus, and the maximum tensile modulus was gained from polycaprolactone/wood flour without MCC [125].

When the NCC was extracted from MCC and added to polyamide 6, the tensile modulus of the composite increased for almost 10 times that of the polyamide 6′s [126]. The montmorillonite (MMT)/NCC reinforcing polylactic acid (PLA) hybrid showed an improvement in the tensile modulus by increasing the load of NCC [127]. When NCC was coated on woven jute/green epoxy composite, enhancement in the tensile modulus occurred [128].

### 4.4. Flexural Modulus

Like all materials, we need to test the resistance of bending of the newly developed material. When microcrystalline cellulose (MCC) was used as a filler to nylon 6, the filler loading improved the flexural modulus from 2.6 GPa with neat nylon 6 to 3.8 GPa with 20% of MCC loading [129]. MCC was also used as reinforcement material to cementitious composites and revealed 106% enhancement in the flexural modulus as a result of adding MCC [130]. The addition of epoxidized citric acid to polylactide/MCC showed the highest result of flexural modulus (4.7 MPa) at 3% of MCC, which was 3.3 MPa before adding MCC [131]. For nanocellulose used as reinforcement of epoxy composite, the addition of NCC increased the flexural modulus until it reached 3.1 GPa at 0.75% of NCC; adding more NCC resulted in the drop of the flexural strength [132]. Poly(lactic acid) (PLA) reinforced with NCC exhibited 106% improvement in terms of flexural strength [133].

### 4.5. Elongation at Break

The deformation that occurs before a material eventually breaks needs to be measured to know the ductility of the material. The addition of MCC extracted from waste-cotton fabric to the hybrid of poly(lactic acid), poly(butylene succinate) revealed acceptable elongation while the other properties were improved [134]. When regenerated MCC was added to epoxidized natural rubber blend film, the elongation was improved by 39% at 20 wt% loading of MCC [135]. Adding NCC extracted from MCC to poly(lactic acid) for packaging applications showed improvement in elongation, reaching 205% [136]. Phosphorylated nanocellulose fibrils added to PVA nanocomposites showed a significant drop in elongation at break encountered with general improvements in the other properties [137].

## 5. Cellulose Aging Resistance

It is known that polymer and plastic materials tend to age under the interaction of oxygen and heat; this aging leads to scrapping [138]. Therefore, resistance to aging is an essential feature of cellulose. The importance of aging resistance depends on the applications that the materials will be used for. Usually, when the thermal and mechanical properties are improved, the aging resistance will also be improved. Enrolling cellulose in polymers gives excellent potential to enhance the polymer’s aging resistance [139,140]. Under normal conditions, the aging resistance can be assessed by tracking the changes in their interfacial shear strength (IFSS) results.

It is clear that adding ash and other non-cellulosic components to the biopolymer reduces the properties of the material, whether these properties are mechanical or thermal, but it has been found that adding ash to the biopolymer improves the aging resistance of the biopolymer. Using cellulose ash as contribution filler improved the aging resistance of the asphalt mixtures from 45.3% to 48.6% [141]. Adding charcoal ash coconut shell to bitumen also improved the aging resistance of the bitumen [142].

The composite of polylactic acid (PLA)/linear low-density polyethylene (LLDPE)/microcrystalline cellulose (MCC) fiber revealed a good aging resistance [143]. The bacterial cellulose used to make electrical insulating paper enhanced its aging resistance [144]. The addition of nanocrystalline cellulose/attapulgite (AT) to polypropylene (PP) improves aging resistance, thermal stability, and gas barrier properties [145]. However, the addition of NCC/AT to PP decreased the mechanical properties and the degradation degree of PP. The composition of nanocrystalline cellulose and potato starch showed improvement in the aging resistance after bleaching the pulps [146]. Comparing the aging resistance of aramid nanofiber (ANF) and nanocrystalline cellulose revealed that ANF has better aging resistance than NCC [147,148]. The NCC boosted the carbon in ionic liquid supercapacitors and improved the aging resistance of the carbon/NCC. This improved the composite’s stability even after three months [149]. Removing moisture and water content exhibited enhancement in the aging resistance of the biopolymer [150]. The nanocrystalline cellulose with copper-based dye-sensitized solar cells demonstrated great improvement in the percentage of aging resistance of the composite [151].

There are three methods to improve aging resistance; these methods are (1) polyhedral oligomeric silsesquioxane, in which some materials can be used to improve the fiber’s resistance, such as modified polyhedral oligomeric silsesquioxane [152]. When poly(p-phenylene benzobisoxazole) nanocomposite fiber was modified by using polyhedral oligomeric silsesquioxane, the aging resistance of poly(p-phenylene benzobisoxazole) was improved [153]. (2) Polyhedral oligomeric silsesquioxanes (POSS) grafting, using 3 aminopropyltrimethoxysilane (APTMS) as a bridging agent. Polyhedral oligomeric silsesquioxanes and silane agent used to graft ZnO nanowires (NWs) onto poly(p-phenylene benzobisoxazole) (PBO) fibers enhanced the aging resistance of the PBO and improved the other properties [154]. (3) Polyhedral oligomeric silsesquioxane (POSS) derivatives in an ionic liquid 1-allyl-3-methylimidazolium chloride (AmimCl). The dispersion of POSS (both aminophenyl or nitrophenyl groups (POSS-AN, NH_2_:NO_2_ = 2:6)) in cellulose matrix, POSS-AN nanoparticles were uniformly dispersed in cellulose at nanoscale, the POSS-AN provided better compatibility in both the AmimCl and cellulose/POSS nanocomposite films and increased the aging resistance [155].

## 6. Comparison between Plant-Derived Nanocellulose and Bacterial Nanocellulose Fibers

**Figure 4:**
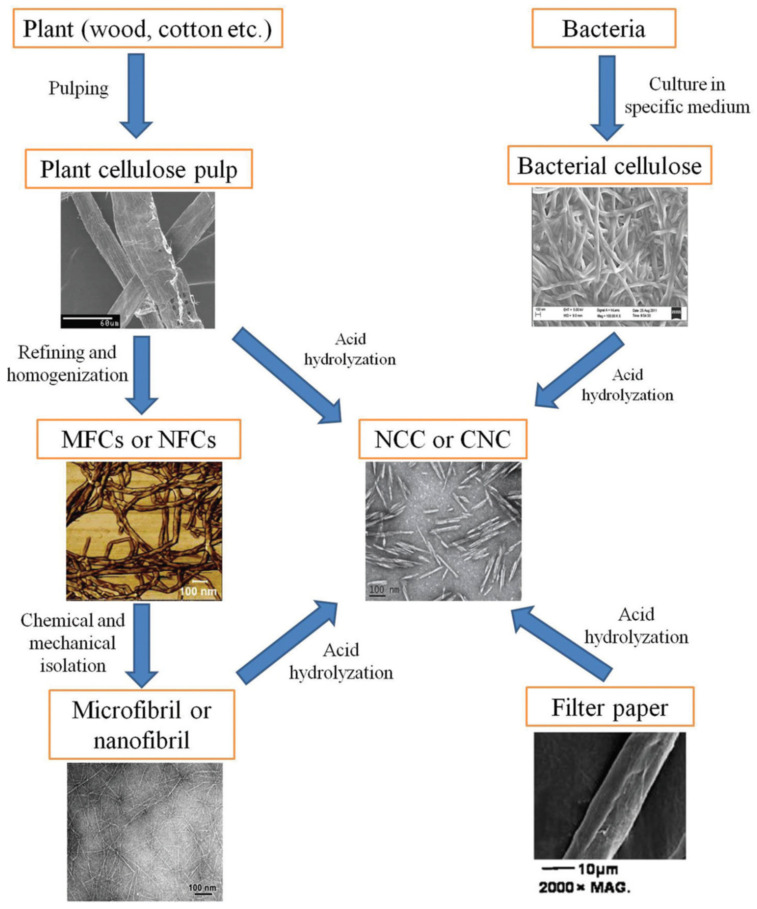
Relationship between different kinds of nanocelluloses [159]. (Reproduced with copyright permission from Creative Commons Attribution License 3.0).

There are differences between plant-based and bacteria-derived nanocellulose fibers. Bacterial nanocellulose possesses higher critical surface tension and higher thermal degradation temperature while the cellulose extracted from plant has hierarchical organization and semi-crystalline nature. The cellulose-based plant is available while the bacterial cellulose is limited [156], and the bacterial cellulose has a higher purity and crystallinity degree than the plant cellulose [157]. Cellulose from plants takes a longer time to be harvested, depending on the plants’ growing duration. Other factors, e.g., plant and soil types, nutrients, climate conditions, and susceptibility to insect pest infestation, also contribute to variances between plant-originated cellulose fibers and bacteria-derived cellulose fibers. The plant-derived cellulose compositions are cellulose, lignin, hemicellulose, and ash, where the nanocellulose extraction process requires energy for harvesting and lignin removal. The drawback of harvesting is that it might cause damage to the environment rather than saving it. On the other hand, the cellulose extracted from bacteria usually needs only days to grow. After cell removal, the cellulose can be extracted in a pure mode. This method demonstrates energy consumption in the sterilization of nutrients and bacterial cellulose and the cell removal processes, which negatively impacts the environment by increasing the air pollution [158]. Figure 4 illustrates the relationship between different kinds of nanocelluloses.

## 7. Applications of Nanocellulose

Applications of any material depend on the appropriateness between the properties of the material and the application specifications and standards. Whenever the properties of any material are improved and are easy to modify, the material will cover more applications. In this case, the improvement in the properties of nanocellulosic materials will extend their applications, where the nanocelluloses applications can be divided by their types. Bacterial nanocellulose’s main applications in the medical field are wound, burn, and ulcer dressings [160]. It is also used in packaging [161], tissue engineering, electronic, optical, sensor, and catalysis applications [162]; whereas NFC and MFC are used as additives in the paper-making process [163], coating [164], medical, pharmaceutical, cosmetic, hygienic [165], barrier material [166], high-temperature thermal insulation [167], and chronic wound healing applications [168]. Because of the small diameter of NCC, it is useful in the medical field applications, especially in the vascular graft [169], electronics, catalysis [170], packaging applications [171,172], synthetic plastic or polymers, fuel cells, filtration, catalysis, tissue engineering, solar cells, and lithium-ion batteries [173]. The NCC bionanocomposites can also be implemented in fire extinguishers [35] and automotive components [174,175] due to their high thermal stability and tensile strength. The applicability of any composites is decided by its controlled durability under the circumstances it is being used. Nanocellulose has a great developing direction on flexible electronics, 3D printing technologies, and smart materials, as well as the medical and energy field applications. Moreover, microcrystalline cellulose is used in many applications, such as abrasives in cosmetics, absorbent, anti-caking, bulking, and aqueous viscosity increasing agents, binder, emulsion stabilizer, slip modifier, and texturizer [176,177].

Designing biocomposite-based cellulose has numerous challenges due to the large variety of cellulosic fibers, polymers, and manufacturing processes, since there are a wide variety of types of reinforcements, dissimilar fiber geometry, and many possibilities for the orientation and fiber arrangement being used especially in large scale design [172]. The sustainability of the environment directly affects the economic development. By increasing the area of cellulose application, the agriculture of cellulose sources will improve, which improves other industries such as agriculture machines industry. Moreover, the development of a growing industry like biocomposites creates more jobs and sub-industries.

## 8. Cellulose Fiber for Injection Molding

Injecting molding is the processing technique (co-extrusion [178] and melt processing techniques [179]) that is used for natural fiber, similar to the synthesis polymers injection methods [180,181]. This method is able to produce parts with very precise dimensions at very low cycle times [182]. The approach used with cellulose is usually the foam injection mold, because it can be used with many different-sized parts and densities [183] to produce a high strength and lightweight molded parts. Many applications require thicker walls of mold than standard injection molding due to producing products effectively. However, the structural foam process allows for a quicker process and lower cycle time on thicker parts and is able to produce complex three-dimensional geometries. Polymeric foams can be prepared by different techniques, such as extrusion, batch, and bead foaming, as well as foam injection molding [184]. Microcellular injection molding technology, also known as the (MuCell) process, is a type of foam injection molding technique that produces parts with excellent dimensional stability using a lower injection pressure [180], which means lower cost. This technique is typically used to produce nanocellulose [185]. Injecting cellulose nanocrystals with biodegradable poly(lactic acid) foam improved the composite properties at a low cost [186]. Microcellular injection molding technology can also be used with cellulose nanofibers. This technique that inject-molded polypropylene foams by introducing hydrophobic-modified cellulose nanofibers has improved the dispersion of cellulose nanofibers in the composite [187]. There are three types of foaming processing technologies.

### 8.1. Batch Foaming Processing

Batch-foaming is a discontinuous foaming process that shows good reproducibility due to the precise process control. It is qualified to be employed to investigate the foaming behavior of polymers and polymer systems and is also used in industrial applications [188]. In this method, the polymer is placed in a high-pressure chamber saturated with inert gas, then, the polymer sample is put under heat and pressure to lower the gas solubility of the polymer [189].

### 8.2. Extrusion Foaming Processing

Foam extrusion is a continuous process of high industrial relevance. It allows producing semi-finished products with foam densities below 50 kg/m^3^ [188]. The temperature must be minimized in this method to avoid emissions during the extrusion foaming processing. This method can be performed with either chemical foaming agents (chemical blowing agents (CBAs)) or physical blowing agents (PBAs) [190,191].

### 8.3. Injection Foam Molding Process

Foam injection molding processing is a composite material produced when a polymer, usually thermoset or thermoplastic, is combined with either an inert physical gas, such as nitrogen, or a chemical blowing agent during the molding process [192]. This method is used to fabricate three-dimensional shapes of polymers using either chemical foaming agents (chemical blowing agents (CBAs)) or physical blowing agents (PBAs) [193,194].

## 9. Nanocellulose Reinforcing Polymer Composite

In general, nanocellulose can be reinforced with synthetic polymer with significantly different chemical and or physical properties when reinforced. Later, the combination of both materials would produce a material with characteristics different from the individual components. The term is usually referred to nanocomposites, which are generally divided into nanocrystalline cellulose (NCC) filled synthetic polymers and nanofabrillated cellulose (NFC) filled synthetic polymers [195]. The addition of nanocellulose has significantly improved synthetic polymer properties, such as tensile strength and thermal conductivity. Synthetizing metal-organic framework (MOF) powders on nanocellulose template offered conventional with excellent mechanical flexibility and porosities as well as shifted the balance of growth and nucleation for synthesizing MOF microcrystals. This low-cost production pathway is capable of transforming MOF into flexible and shapeable form and thus range their applications in more wide fields [196]. Table 1 exhibits some examples of nanocrystalline cellulose-filled synthetic polymer and the effect of nanocellulose reinforcement.

The issue of reinforcement of nanocrystalline cellulose with synthetic polymer has received considerable critical attention. Roohani et al. [197] studied the influence of NCC contents on the morphological, dynamic mechanical, and tensile properties of cotton NCC reinforced with copolymers of polyvinyl alcohol and polyvinyl acetate. For dynamic mechanical analysis results, glass transition temperature rose significantly with the addition of cotton NCC due to the formation of a water layer at the interface, which caused its matrix to become less plasticized by water. On top of that, the tensile strength and Young’s modulus seemed to increase with the addition of cotton NCC. However, the humidity elevation caused a significant decrease in the tensile modulus due to the glass transition temperature changes towards lower values, below the room temperature. Cao et al. [198] also looked at the effect of NCC content but with differences in NCC sources and its polymer matrix. They studied the NCC contents of flax fillers and tested for tensile, dynamic mechanical, and thermal gravimetric properties of flax NCC to reinforce nitrile composites. The composites performed an increase of tensile strength and storage modulus with the increase of filler content. Moreover, the rise in flax NCC also resulted in glass transition temperature (*T_g_*) of the composites that was shifted from 10.8 to 17.2 °C. In terms of thermal gravimetric analysis, the degradation temperature corresponding to flax NCC in nanocomposites was significantly higher than pure flax NCC.

In addition, Mahendra et al. [199] carried out a study on the effect of oil palm NCC and TEMPO-oxidized nanocellulose on the compatibility of polypropylene/cyclic natural rubber (PP/CNR) blends. The result showed that the addition of NCC enhanced the mechanical properties of the polymer nanocomposites compared to the neat polymer. Moreover, the improvement of NCC nanocomposites was also observed as the result of interphase surface tension and thermal stability. A recent study was carried out by Dai et al. [200] on the potential of the green method to fabricate green pH/magnetic sensitive hydrogels based on pineapple peel crystalline nanocellulose (rPPNc) and polyvinyl alcohol. The rPPNc improved the thermal stability, swelling ability, naringin loading, and entrapment efficiency of the hydrogels. A study conducted by Jain and Pradhan [201] stressed that the mechanical properties of sisal NCC-rubber composites stress strain graphs displayed a ductile fracture behavior, where a peak yield stress occurred followed by necking and cold drawing. The increase of sisal NCC fillers within 5–10% in the nanocomposites would increase about 0.365 to 0.360 MPa compared to pure rubber. The process of extraction of NCC fillers from sisal leaves in this research is the acid hydrolysis method. However, the tensile strength of sisal nanocomposite was less than the sisal fiber composite due to the lack of homogeneity in mixing sisal NCC in latex and weak bonding between cellulose and latex.

There have been several investigations into the causes of the effect of nanofibrillated cellulose when reinforced with polymer. Karmaker et al. [202] implemented solution casting to fabricate PVA-gelatin films with the addition of NCC fillers from softwood kraft pulp. The addition of nanofiller showed drastic changes with its mechanical and thermal properties. Those tensile moduli and strength were significantly increased as gelatin and NCC fillers were added in the nanocomposite, which contributed to low elongation at break. Moreover, the addition of NCC filler in the PVA-gelatin nanocomposites reduced the moisture absorption as their thermal properties was improved. The surface morphology of the nanocomposite permitted better crystallinity due to the existence of glycosidic bonds in cellulose structure. Another study executed by Xu et al. [203] found that tunicin cellulose had potent effects in terms of mechanical and thermal properties in epoxy nanocomposites. The storage modulus and *T_g_* of the nanocomposites were significantly enhanced when the increase of tunicin NCC filler. For instance, with 15 wt% of tunicin NCC fillers, the storage modulus was increased by 100% relative to pure epoxy, while their *T_g_* increased to 75.5 °C. Moreover, the inclusion of NCC in epoxy nanocomposites tremendously increased the tensile strength up to 60 MPa due to good surface adhesion NCC filler with epoxy matrix. In addition to that, another well-known nanofiller is nanofibrillated cellulose (NFC). It is commonly used in packaging and automotive applications due to its enhanced thermal, mechanical, and crystallinity properties. Table 2 summarizes the effect of NFC in synthetic polymer matrix composites.

As mentioned by Zhang et al. [204], bagasse pulp NFC was blended with γ-aminopropyltriethoxysilane treated aluminum nitride nanosheets (TAlN). The results found that dispersibility of the AlN nanosheets in the NFC substrate was enhanced because of the silane treatment. This happened due to the treatment that lowered scattering between the AlN and NFC interfaces, which induced better thermal conductivity. The new material has shown enhancement of the mechanical properties of nanocellulose reinforced synthetic polymer composites. Pandurangan and Kanny [205] evaluated morphological and curing properties of banana NFC-filled epoxy composites. They stressed that the banana NFC fillers acted as a catalytic curing agent by increasing the cross-link density during gelation of epoxy. Moreover, good dispersion of the banana NFC particles in the matrix contributed to 10% increase in tensile strength and 26% increase in elongation at 3 wt% of NFC filled epoxy nanocomposite. Along with the mechanical properties, nanocomposite film’s dynamic mechanical properties were improved, especially at 2–3 wt% of NFC filler. Water uptake results suggested that the water uptake of the NFC filled epoxy nanocomposites was reduced as with higher concentration of NFC particles. The same study was also conducted by Nair et al. [207] by using western red cedar NFC with high residual lignin. They found a significant increase in terms of mechanical, thermal, and water barrier properties of the high residual lignin NFCs nanocomposite.

Recently, studies on nanocellulose reinforced epoxy have been performed by researchers in many fields such as aerospace, automotive, and marine construction [205,206,207,208]. Vu et al. [208] carried out a study on the influence of micro/nano white bamboo fibrils on the physical characteristics of epoxy resin reinforced composites. They found that the inclusion of the NFCs increased the flexural and tensile behaviors, fracture toughness, and thermal properties of the nanocomposite. The presence of white bamboo NFCs enhanced the tensile and flexural moduli, which exhibited improvement in the nanocomposite’s stiffness. Another study by Junior et al. [209] also looked at the impact of the nanofibrillation of bamboo pulp but with a slight difference in focus from Vu et al. [208]. They studied the nanocomposite based on starch/PVA blend and tested for thermal, structural, and mechanical properties. The results indicated that the higher NFC filler encouraged better homogeneity, cohesion, and more compact structure, which promoted larger crystals in the nanocomposite. In addition, the tensile strength and elongation at break improved at 24 and 15% as compared to the control blend.

## 10. Nanocellulose Reinforcing Biopolymer Composite

As nanocellulose is used to reinforce synthesis polymer, it is also employed to reinforce biopolymer from natural resources to improve the properties of the natural polymer, such as polylactic acid (PLA), polyhydroxy acids (PHA), polyhydroxybutyrate (PHB), polybutylene succinate (PBS), and starch biopolymer [210,211]. Table 3 and Table 4 illustrate some examples of NCC and NFC reinforced biopolymer and show the effects of NCC as well as NFC fillers.

Vaezi et al. [226] evaluated the effect of coatings on bionanocomposites of cationic starch/cotton NCC in the application of paper packaging. They discovered that the increase in NCC nanoparticle loading increased the tensile strength, oil resistance, and air resistance of the coated paper, with the optimized amount of 5 wt% of NCC nanoparticles. In terms of physical properties, the water absorption of the coated paper decreased by 50% at 5 wt% NCC concentration. Jiang et al. [227] performed a study regarding the impact of eggshell NCC filler on the overall properties of corn starch films. They showed that the inclusion of the fillers noteworthily boosted the tensile behavior, thermal stability, oxygen, and water vapor barrier properties compared to the pure corn starch film. This happened due to the inductive effect between the C–C bonds on cornstarch skeleton and the O–C–O bond on calcium carbonate in the eggshell nanofiller, which later contributed to strong interaction and biocompatibility between the two components.

Another well-known source of NCC filler reinforced in biocomposites is orange peel. It has been widely used in the packaging industry in order to develop high durability biofilm. A study conducted by Fath et al. [228] found that orange peel NCC filler had good dispersion in their matrix. The filler also acted as a compatibilizer, which improved the physical interaction between NCC filler-starch films. The inclusion of the fillers also decreased the water vapor transmission rate, especially at 2 wt% concentration. A research study carried out by Nasution et al. [229] measured the effect of filler loading and co-plasticizer addition on rattan NCC-filled sago starch bionanocomposite. They stressed that the lowest water absorption rate was 9.37% at an additional of 3 wt% rattan NCC filler and 10 wt% acetic acid as compared to pure films.

For the effect of acetyl treatment of starch NCC filler to overall properties of the PLA based nanocomposites, the nanocomposites of 1 and 3 wt% fiber loadings of untreated and treated fiber as well as control were tested for the morphological, barrier, and mechanical properties [212]. It was observed that the PLA nanocomposites with treated starch NCC filler provided better filler dispersion and interaction with the matrix. Moreover, it also had a high potential to improve the oxygen barrier and tensile properties of PLA nanocomposite. Syafri et al. [230] studied the influence of sonication time on thermal stability, biodegradation, and moisture absorption performance of water hyacinth NCC/bengkuang starch bionanocomposites. The result showed that the bionanocomposite vibrated for 60 min had the highest thermal stability and presented with low moisture absorption capability. Furthermore, the 60 min-vibrated nanobiocomposite had low porosity formation and a coarse surface. The bionanocomposite also had a slow biodegradation rate, which is highly suitable for the application of food packaging bags. As well as NCC-filled bionanocomposites, NFC filler can be implemented in a biopolymer matrix to form durable composite films. Table 4 illustrates some examples of NFC-filled biopolymer composite and shows the effect of NFC fillers.

A study conducted by Lomelí-Ramírez et al. [250] studied the mechanical properties of dried and hydrated *Agave tequilana* Weber NFC filled in corn starch bionanocomposite. It showed tremendous improvement in terms of tensile, flexural, and impact performance due to a small amount of 1 wt% of NFC filler. This could be attributed to an increase of stiffness caused by NFC filler in the bionanocomposite. Pitiphatharaworachot et al. [251] evaluated the bamboo holocellulose NFC fillers reinforced in thermoplastic starch (TPS) bionanocomposites. The NFC filler was prepared from bamboo holocellulose powder using TEMPO-mediated oxidation. They established that bamboo NFC filler was individually dispersed with TPS matrix, which subsequently contributed to less water uptake, high transparency, better tensile strength, and better modulus as compared to pure TPS films. The optimum concentration of NFC filler was 1.5 wt%.

In accordance with Huang et al. [221], they carried out research to compare the effects of various modification methods (silane and malic acid treatment) on physical and chemical properties of cassava-filled cassava starch bionanocomposites. In their findings, modified NFC filler significantly improved dispersibility with those fibrils that were detached from each other. Morphologically, they formed three-dimensional network structures with no occurrence of coarse fiber aggregation. The inclusion of modified cassava NFC filler improved the tensile strength, hydrophobicity, and water vapor transmission coefficient of the bionanocomposite films by 1034%, 129.4%, and 35.95%, respectively.

Furthermore, De Almeida et al. [253] performed a study on thermal, physical, and mechanical behaviors of regular and waxy corn starch films reinforced with eucalyptus NFC filler. The moisture content, water solubility, and water vapor permeability were significantly reduced by the presence of NFC filler for both regular and waxy starch films. It was possible to observe that the addition of NFC filler enhanced the thermal and tensile properties of the bionanocomposite since only 1% of the suspension was added. Balakrishnanan et al. [254] studied the effect of filler loading on morphological, transport property, and viscoelastic polymer chain confinement of pineapple leaf NFC filled in thermoplastic potato starch bionanocomposites. They confirmed that the polymer chain confinement around the NFC filler has excellent dispersion and superior interaction between matrix and NFC filler. The bionanocomposites obey the pseudo-fickian property. In terms of barrier properties, the addition of NFC filler at 3 wt% concentration also resulted in its enhancement. However, further increased filler content would depreciate the properties due to the agglomeration of fiber.

Another study by Tajik et al. [255] evaluated the impact of cationic starch in the presence of NFC filler on the structural, optical, and strength properties of paper. They found retention and reinforcing effects of the additives on the paper network. In this manner, the mechanical properties such as tensile and burst strengths were drastically increased as the increasing levels of the additives were up to 33% and 23%, respectively, for 0.6 CS/2% NFCs filler paper bionanocomposites. The bagasse NFCs filler also contributed to the improvement of retention and drainage of pulp at lower levels because of the interaction between filler and starch polymer. In addition, a higher concentration of the nanofillers improved the brightness of paper. Research work by Soni et al. [256] blended TEMPO-oxidized NFCs fillers inside three modified starches, namely hydroxypropyl starch (HPS), acetyl starch (AS), and acetyl oxidized starch (AOS), to evaluate their mechanical strength and durability in water. They discovered that TNFC/acetyl oxidized starch biofilm displayed less water swelling and improved wet tensile properties due to the formation of hemiacetal between nanofiller and starch polymer. TNFC/HPS biofilm illustrates the highest wet stiffness with the minimum swelling in water.

## 11. Conclusions

There is no escape from stopping or at least minimizing the usage of non-degradable petroleum-sourced materials to protect the environment. Micro- and nanocellulose are good alternatives to manufacture composites with either natural starch or synthetic matrix. The cellulose classification and extraction methods have been highlighted. The development of composite materials containing cellulose has improved in terms of mechanical, thermal, and aging resistance. Further improvements to enhance the dispersion and compatibility of cellulose have been discussed. These advancements have been highlighted to reveal the high potential of cellulose-based composite for a large number of applications. The increment of using plastic materials in the world requires more development in bioplastic materials to replace petroleum-based synthesis polymers. Processes of extracting, isolating, and injecting cellulose require more studies and adjustments to improve the properties of the biopolymers and to have broader applications.

## Figures and Tables

**Table 1:** Illustration of the effect of nanocrystalline cellulose on synthetic polymer matrix.

Source of Nanocrystalline Cellulose	Synthetic Polymer	The Effect and Advantages of the Reinforcement	Ref.
Cotton	Copolymers of polyvinyl alcohol and polyvinyl acetate	- Improvement of dynamic mechanical, tensile strength, and Young’s modulus as NCC content increased. - Glass transition temperature rose significantly as the addition of cotton NCC. - Elevation of humidity resulted in significant declining of the tensile modulus.	[197]
Flax	Nitrile rubber	- Increase of tensile strength (7.7–15.8 MPa) and storage modulus. - Higher thermal degradation temperature. - T_g_ of the nanocomposites was shifted 10.8 to 17.2 °C with flax NCC content increasing to 20 phr.	[198]
Oil palm	Polypropylene/cyclic natural rubber	- Improvement of interphase surface tension and thermal stability. - More homogenous than without the addition of the nano-fillers.	[199]
Pineapple	Polyvinyl alcohol	- The nanocomposites are used to develop green pH/magnetic sensitive hydrogels. - Improvement in terms of thermal stability, swelling ability. - Significant enhancement of naringin loading and entrapment efficiency of the hydrogels.	[200]
Sisal leaf	Rubber	- Increase in tensile properties of the nanocomposites.	[201]
Softwood Kraft pulp	Poly Vinyl Alcohol	- The gelatin and NCC fillers help to increase the tensile strength and Young’s modulus. - The moisture absorption of NCC/gelatin/PVA nanocomposites tremendously increased as compared to PVA films. - Thermal properties such as thermal degradation and glass transition temperature enhanced. - Better crystallinity due to the existence of glycosidic bonds in cellulose structure.	[202]
Tunicin	Epoxy	- Improved tensile strength due to good adhesion with epoxy matrix. - Provided better dynamic mechanical properties in their synthetic polymer.	[203]

**Table 2:** Illustration of the effects of nanofibrillated cellulose on synthetic polymer matrix.

Source of Nanofibrillated Cellulose	Synthetic Polymer	The Effect of the Reinforcement	Ref.
Bagasse pulp	Aluminium nitrite	- The effect of silane treatment along with NFC substrate improved the thermal conductivity. - The composite film is highly suitable for green electronic devices applications.	[204]
Banana	Epoxy	- Reduce the water uptake of nanocomposite film, especially at 5 wt% of NFC. - The mechanical and dynamic mechanical properties significantly improved at 2–3 wt% of NFCs. - Act as a catalytic curing agent.	[205]
Northern bleached softwood kraft (NBSF) pulp	Epoxy	- Improved the nanocomposite storage modulus as well as their tan δ. - Tensile and flexural properties significantly increased as the inclusion of NFC fillers. - Thermal stability and residual char of kenaf/epoxy composites was well enhanced.	[206]
Western red cedar	Epoxy	- Strong reinforcing effects displayed by the high residual lignin containing NFCs on the mechanical, physical, thermal properties of the nanocomposite. - High residual lignin of NFC provided impermeable medium for moisture in epoxy composites.	[207]
White bamboo	Epoxy	- Improvement in tensile and flexural properties, fracture toughness, as well as thermal property especially at 0.3 wt% of NFC. - Better in dynamic mechanical properties in both tensile and bending condition as the addition of NFCs fillers at 0.3 wt%.	[208]
Bamboo	Starch/PVA	- Better homogeneity, cohesion, and more compact structure, which promotes larger crystals in the nanocomposite. - Tensile strength and elongation at break improved at 24 and 15% as compared to the control blend.	[209]

**Table 3:** Illustration of the effect of nanocrystalline cellulose on biopolymer matrix.

Source of Nanocrystalline Cellulose	Source of Biopolymer	The Effect of the Reinforcement	Ref.
Maize starch	Polylactic acid	- Addition of starch NCC filler showed the PLA nanocomposite to have high potential to improve the oxygen barrier and tensile properties. - Provided better filler dispersion and interaction with the matrix.	[212]
Nata-de-coco	Polylactic acid	- Enhancement in viscoelastic properties up to 175% in terms of storage modulus in bending. - Addition of 2 wt% nanocellulose into PLA resulted in moderate strength improvement.	[213]
Bamboo pulp	Polylactic acid	- PLA-grafted NCC (PLA-g-NCC) films display uniform dispersion of NCC due to the efficient grafting, results in enhancement in tensile strength. - The elastic and crystallinity properties of the nanocomposites improved with increasing of NCC loadings.	[214]
Coffee silver skin	Polylactic acid	- NCC with loading of 3 wt% in PLA film enhanced water barrier and mechanical properties of nanocomposites. - Nanocomposite with NCC can overcome drawbacks of biopolymer film.	[215]
Microcrystalline cellulose	Polylactic acid	- NCC-reinforced PLA exhibited improvement in thermal, mechanical, and UV barrier properties.	[216]
Microcrystalline cellulose	Polylactic acid	- The reinforcement of the polyethylene glycol (PEG) and NCC improved the crystallinity of the PLA. - The impact and the elongation at break increased from 0.864 to 2.64 kJ, and 22 from 11% to 106.0%, respectively.	[217]
Microcrystalline cellulose	Polylactic acid	- The addition of NCC into the PLA showed an increment on tensile strength of PLA and PLA-g-silane nanofiber. - The modified PLA nanocomposite considered as a practical candidate for hard tissue engineering applications according to cytotoxicity results.	[218]
Microcrystalline cellulose	Polylactic acid	- Acetylation can improve the performance of the composite by enabling linkages between carbonyl groups, helping to establish a good stress transfer between the fiber and the matrix.	[219]
Nanocrystalline cellulose	Polyhydroxy acids	- Nanocellulose-reinforced PHA films improved the mechanical properties by 23% compared to neat PHA samples. - Increase of the crystallinity and stiffness of the nanocomposites. - Surface roughness of the nanocomposites was increased, which contributed to better interlaminar bonding in multi-layer composites applications. - Presence of UV blocking effect.	[220]
Kenaf	Polyhydroxy acids	- Improve the conductivity of the polymer nanocomposites.	[221]
Bleached pulp board	Polyhydroxybutyrate	- Nanocellulose worked as heterogeneous nucleating agent in PHB. - Crystallinity of polymer was reduced and improved the toughness of PHB. - The mechanical properties of the nanocomposites such as Young’s modulus and elongation at break increased by 18.4% and 91.2%, respectively.	[222]
Nanocrystalline cellulose	Poly(3-hydroxybutyrate-co-3-hydroxyvalerate) (PHBV)	- NCC was dispersed evenly in GMA-g-PHBV. - Limited reinforcement observed despite enhanced dispersion relative to the neat PHBV matrix due to the hydrophobization surface of NCC.	[223]
Nanocrystalline cellulose	Polybutylene succinate	- Restricted the mobility of polymer chains and promoted nucleation and recrystallization of polymer. - Degree of crystallinity increased from 65.9 to 75.6%. - The tensile strength increased from 23.2 MPa to 32.9 MPa. - Oxygen transmission rate of PBS films was decreased from 737.7 to 280 cc/m^2^/day. - Water vapor transmission rate (WVTR) of PBS films decreased from 83.8 to 49.4 g/m^2^/day.	[224]
Microcrystalline cellulose (MCC)	Poly(butylene succinate) (PBS)/polylactic acid (PLA)	- Impact strength, moduli, and crystallinity of the nanocomposites increased. - Thermal stability, storage modulus, glass translation temperature of nanocomposites increased.	[225]
Cotton	Cationic starch	- Increased in tensile strength, oil, and air resistance of the coated paper composites with the optimized amount for the NCC nanoparticles was 5 wt%. - Water absorption of the coated paper composite decreased by 50% at 5 wt% NCC concentration.	[226]
Eggshell	Corn starch	- The eggshell nanofiller was uniformly dispersed and reinforced within film matrix. - Tensile properties, thermal stability, water vapor, and oxygen barrier properties were also tremendously improved as compared to pure starch film.	[227]
Orange peel	Starch	- Significant improvement of water barrier properties at 2 wt% concentration. - Well dispersed in starch matrix during formation of biofilms. - Act as compatibilizer.	[228]
Rattan biomass	Sago starch	- Rattan NCC filler decreased the water uptake of the bionanocomposite.	[229]
Water hyacinth	Bengkuang (*Pachyrhizus erosus*) starch	- 60 min vibrated water hyacinth NCC/bengkuang bionanocomposites had slow biodegradation rate. - The optimum 60 min vibrated samples resulted in high thermal stability and low moisture absorption rate.	[230]
Sugar palm fiber	Sugar palm starch	- Improvement in the water barrier property and water vapor permeability (WVP) of the nano composite film by 19.94%. - Improvement in mechanical, thermal, and physical properties.	[231,232]
Kenaf fibers	Cassava starch	- Enhancement in the tensile strength and modulus of the biocomposite films. - Decreased the water absorption by the biocomposite or the water sensitivity.	[233]
Garlic stalks	Corn starch	- Scanning electron micrographs of the films showed homogeneous dispersion of nanocrystalline cellulose in the starch matrix. - Improvement in tensile strength and modulus and improvement in moisture property.	[234]
Kenaf fibers	k-carrageenan	- The biocomposite showed enhancement in mechanical properties and thermal stability. - The biocomposite film shows a good dispersion of the cellulosic fiber on the starch matrix.	[235]
Sugarcane bagasse fiber	Maize starch	- Improvement in water vapor barrier properties with addition of nanocrystalline cellulose.	[236]
Cotton cellulosepowders	Plasticized starch	- Improvement in the thermal stability, mechanical properties, and air permeability.	[171]
Potato peelwaste	Potato starch	- Enhancement in tensile modulus and water permeability property.	[237]
Sugarcane Bagasse	Tapioca Starch	- The nanocellulose was found in good dispersion in starch-based tapioca biocomposite. - Resulting in good adhesion bonding. - Improved tensile strength up to 20.84 MPa with the incorporation of 4% nanocellulose.	[238]

**Table 4:** Illustration of the effect of nanofibrillated cellulose on the biopolymer matrix.

Source of Nanofabrillated Cellulose	Biopolymer	The Effect of the Reinforcement	Ref.
Kenaf pulp	Polylactic acid	- The tensile properties of nanocomposites indicated that strength and modulus were improved with increasing NFC contents.	[239]
Banana waste	Polylactic acid	- The incorporation of 20 wt% of glycerol triacetate and 1 wt% of nanocellulose doubled the degree of crystallinity. - Dynamic mechanical thermal analysis (DMTA) exhibited a 30 to 50% reduction in storage modulus (stiffness) when compared to neat PLA.	[240]
Nata-de-coco	Polylactic acid	- The tensile modulus of the laminated nanocellulose composites was found increasing (from 12.5–13.5 GPa), insensitive to the number of sheets of nanocellulose in the composites. - Tensile strength of the laminated nanocellulose composites decreased by 21% (from 121 MPa to 95 MPa) when the number of reinforcing nanocellulose sheets increased from 1 to 12 sheets.	[241]
Linter pulp	Polylactic acid	- The impact strength, tensile strength, and Young’s modulus of nanocomposites (PLA/CNF5/PLAgMA5) increased by 131%, 138%, and 40%, respectively, compared to neat PLA, with increasing of nanocellulose.	[242]
Kenaf	Polylactic acid	- The strength and tensile modulus increased from 58 MPa to 71 MPa, and from 2.9 GPa to 3.6 GPa, respectively, for nanocomposites with loading of 5 wt% NFC. - The storage modulus of the nanocomposites increased compared to neat PLA. - The addition of NFC shifted the tan delta peak towards higher temperatures. - The tan delta peak of the PLA shifted from 70 °C to 76 °C for composites with 5 wt% CNF.	[243]
Carrot pomace	Polylactic acid	- The incorporation of nanocellulose increased hydrophilicity. - The transmission rates of oxygen, carbon dioxide, and nitrogen increased after incorporating nanocellulose into PLA.	[243]
Bleached birch Kraft pulp	Polyhydroxyalkanoates (PHA)	- The reinforcement of nanocellulose with polymers improved mechanical properties, water contact resistance, and higher barrier performance against water vapor compared to the neat nanopapers.	[244]
Ethyl cellulose	Poly(ethylene glycol)dimethacrylate	- Improved the compressive strength of nanocomposites.	[245]
Bleached pulp board	Polyhydroxybutyrate (PHB)	- The light transmittance, tensile strength, and elongation at break were reduced. - The crystallinity, thermal properties, and Young’s modulus were increased.	[222]
Bleached Kraft eucalyptus fibers	poly (3-hydroxybutyrate-co-3-hydroxyvalerate, PHBV)	- Incorporation of nanocellulose increased tensile modulus, thermal degradation, and storage modulus. - Nanocellulose promotes the early onset of crystallization. - Inhibit foaming. - Decreased the solubility of CO_2_ and increased desorption diffusivity.	[246]
Regenerated cellulose	poly(3 hydroxybutyrate) (PHB)	- Increased loading of regenerated cellulose decreased the tensile strength and elongation at break.	[247]
Nanofibrillated cellulose	Polybutylene succinate (PBS)	- Incorporation of nanocellulose has drastically increased the crystallinity of nanocomposites, thus acting as nucleating agents. - Form flexible nanocomposite films. - Improve the mechanical properties of nanocomposite films.	[246]
Wood cellulose pulps	Chitosan	- The mechanical properties and thermal stability of chitosan nanocomposite foams increased. - The chitosan nanocomposite foams displayed a highly efficient water/oil separation capacity even at 90 °C. - Goof biocompatibility with L929 mouse fibroblasts.	[248]
Bleached pine sulfite dissolving pulp	Chitosan	- The incorporation of NFC improved the mechanical properties of composites of chitosan hydrogel matrices.	[249]
*Agave tequilana* Weber	Corn starch	- Drastic improvement in term of tensile, flexural, and impact performance.	[250]
Bamboo helocellulose	Thermoplastic starch	- Bamboo NFC filler well-dispersed in TPS matrix which contributed high in tensile properties. - Lesser in water uptake of bionanocomposite.	[251]
Cassava residue cellulose	Cassava starch	- Improved the tensile strength, hydrophobicity, and water vapor transmission coefficient of the bionanocomposite films by 1034%, 129.4%, and 35.95%, respectively. - Improved dispersibility with those fibrils that were detached from each other.	[252]
Eucalyptus	Waxy corn starch	- The moisture content, water solubility, and water vapor permeability were significantly reduced by the presence of NFC filler for both regular and waxy starch films. - Thermal and tensile properties also increased at only 1% of suspension.	[253]
Pineapple leaf	Thermoplastic potato starch	- Polymer chain confinement around NFC filler had excellent dispersion and superior interaction between matrix and NFC filler. - Barrier properties were enhanced.	[254]
Softwood alpha cellulose pulp	Cationic starch	- Improved the tensile and burst strengths of the paper composites. - Contributed to enhancement of retention and drainage of pulp paper due to interaction between fillers and polymers. - Improved brightness of paper.	[255]
Softwood cellulose pulp	Modified starch	- The implementation of acetyl oxides starch with TEMPO-oxidized cellulose nanofibre (TNFC) filler resulted in less swelling inside water and highest wet tensile behaviors.	[256]
Kenaf fibers	Maize starch	- Addition of nanofibrillated cellulose to the starch enhanced the mechanical properties (in terms of tensile strength and Young’s modulus) and the thermal stability of the nanocomposites. - Reduced moisture absorption. - Decreased water sensitivity.	[257,258]
Bamboo nanofibers	Cassava starch	- The nanofibrillated cellulose increased tensile strength of 50% of starch films, while the elongation at break showed similar increase (66%) at concentration of 1.0 g/100 g of nanofibrillated cellulose. - Improvement in the structure of the composite.	[259]
Rice straw	Potato starch	- The yield strength and Young’s modulus of the nanocomposite enhanced after adding the nanofibrillated cellulose to the starch. - The glass transition temperature increased. - The humidity absorption resistance of films was significantly enhanced by using 10 wt% cellulose nanofibers. - The transparency of the nanocomposites was reduced compared to the pure starch composite.	[260]
Sugar palm	Sugar palm starch	- Improved water absorption and water solubility properties of the nanocomposite films by 18.84% and 39.38%, respectively. - Good compatibility between the nanofibrillated cellulose and the sugar palm fiber, the composition created intermolecular hydrogen bonds between them.	[261,262,263,264]

## Data Availability

The data presented in this study are available on request from the corresponding author.
